# Insights to Clinical Use of Serial Determination in Titers of Cyclic Citrullinated Peptide Autoantibodies

**DOI:** 10.1155/2007/12367

**Published:** 2007-03-07

**Authors:** Toshiaki Kogure, Takeshi Tatsumi, Hiroshi Fujinaga, Atsushi Niizawa, Katsutoshi Terasawa

**Affiliations:** ^1^Department of Integrated Japanese Oriental Medicine, School of Medicine, Gunma University, Maebashi, Gunma 371-8511, Japan; ^2^Division of Japanese Oriental Medicine, Department of Internal Medicine, Toyama Prefectural Central Hospital, 2-2-78 Nishinagae, Toyama 930-8550, Japan; ^3^Department of Japanese Oriental Medicine, Kanebo Memorial hospital, Kobe, Hyogo 652-0855, Japan; ^4^Department of Japanese Oriental Medicine, Graduate School of Medicine, Chiba University, Chiba 260-8670, Japan

## Abstract

Anti-cyclic citrullinated peptide (CCP) antibody is a useful marker for the diagnosis and prognosis of rheumatoid arthritis (RA). Recently, clinical significance of follow-up in anti-CCP antibody titer has been pointed out. Thus, we investigated the serial determination in anti-CCP antibodies titer in RA patients. Six patients with RA, who were followed up for longer than 5 years, were assessed in anti-CCP antibodies and radiographs (Larsen score). Anti-CCP antibodies in frozen sera were measured using ELISA. As a result, 6 patients with RA were divided into two groups: one possessed high titers without variation, and the other was without high titers. Joint damage progressed during observation in 2 out of 3 patients with high anti-CCP titers in a retrospective assessment. In contrast, the RA patient, whose anti-CCP titer decreases although it had been high titer at baseline, did not show increase in the Larsen score. These findings suggest that it might be necessary to analyze changes in anti-CCP to predict the prognosis of joint destruction.

## 1. INTRODUCTION

Rheumatoid arthritis (RA) is a chronic inflammatory autoimmune disease characterized by progressive cartilage erosion and destruction. Previous investigations have shown that autoantibodies to a cyclic citrullinated peptide (anti-CCP) are highly specific for RA, including the early form, and that these antibodies may be of prognostic value as markers predicting progression to more serious disease [1–3]. Recently, it has been reported that serially determined anti-CCP performs better than baseline determination for predicting radiographic progression in patients with RA [4]. Therefore, we retrospectively investigated the association between variations in anti-CCP titers and the progression of joint damage in our RA patients, who had not been treated with anti-TNF-alpha drugs and tacrolimus hydrate.

## 2. PATIENTS AND METHODS

**Figure 1:**
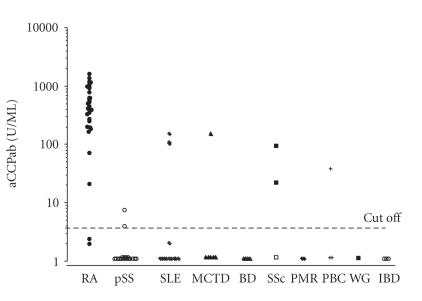
Anti-CCP titer in RA (rheumatoid arthritis: 23 females and 8 males) who fulfilled the diagnostic criteria for RA or other various autoimmune diseases. Autoimmune diseases except for RA, such as primary Sjogren's syndrome (PSS; *n* = 12), systemic lupus erythematosus (SLE; *n* = 13), mixed connective tissue disease (MCTD; *n* = 6), Bechet's disease (*n* = 4), systemic sclerosis (SSc; *n* = 3), polymyalgia rheumatica (PMR; *n* = 2), primary biliary cirrhosis (PBC; *n* = 3), Wegener's gnanuloma (WG; *n* = 1), and inflammatory bowel disease (IBD; *n* = 3), were also assessed. Two patients with SSc overlapped with RA.

Firstly, to confirm the specificity and sensitivity of anti-CCP antibodies in our hospital, we assessed anti-CCP titer in RA or other various autoimmune diseases (see Figure 1) before serial determination in RA.

In the longitudinal observation, anti-CCP antibody was detected in sera obtained from 6 RA patients who were followed up for 5 years. Each serum sample was frozen at −80°C and stored. The 6-patient profiles at the start of follow-up are demonstrated in Table 1. They fulfilled the diagnostic criteria for RA. Radiographic assessment was performed retrospectively. Serial radiographs of the hands and feet (standard film on anteroposterior projection) were taken from the start until year 5 during regular clinical assessments. The radiographs were evaluated according to Larsen and Dale [5] and Lindqvist et al. [6]. Anti-CCP antibodies were assessed with a commercial enzyme-linked immunosorbent assay (ELISA: second generation; Axis-Shield Diagnostics Limited, UK), according to the manufacturer's instructions. In brief, serum samples were diluted 1 : 100, or more for cases in which the antibody level was very high with optical densities not falling within a standard curve at the original dilution. The samples were incubated for 60 minutes at room temperature. After incubation, each well was washed with washing buffer 3 times. One hundred ul of substrate were added to each well. After a 30-minute incubation, each sample was measured for its absorbance at 550 nm. Each assay was carried out in duplicate.

## 3. RESULTS AND DISCUSSION

Anti-CCP titers in patients with RA or other autoimmune diseases are shown in Figure 1. Anti-CCP was positive in 94.7% of the patients with RA and negative in 88.8% of the patients with other autoimmune diseases except for RA. These results are in accordance with the previous report [1]. Two systemic sclerosis (SSc) patients with anti-CCP were complicated with RA.

**Figure 2:**
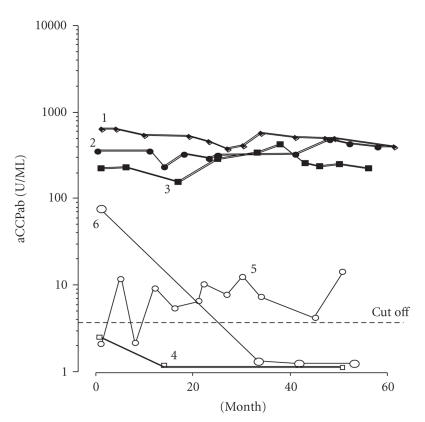
Anti-CCP titer changes in each patient. The numbers refer to patient no. 5 in Table 1.

Anti-CCP titers changes in the 6 patients are shown in Figure 2. Three of the patients (no. 1, 2, and 3 in Table 1) showed a high anti-CCP titer and one patient (no. 4) showed a low titer over 5 years, without variation. In serum obtained from patient no. 5, anti-CCP was not detected during the disease course. Although anti-CCP titer was high at the start of observation in patient no. 6, anti-CCP was not detected in her sera during the succeeding four years.

**Figure 3:**
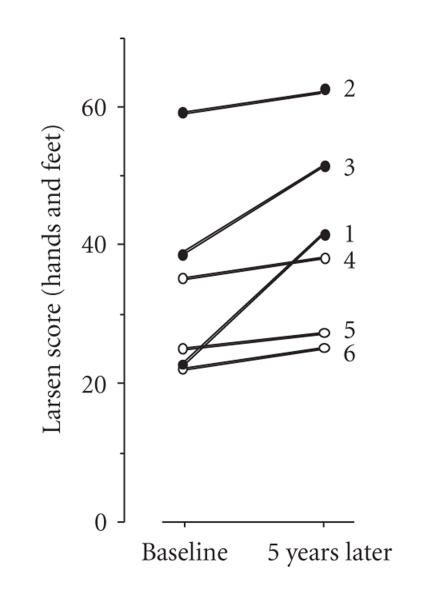
The change in Larsen score during observation. Closed circle (•) indicates the patients with serial high titers in anti-CCP. The numbers refer to patient no. 5 in Table 1.

We show the variations in radiographic damage in each patient according to Larsen and Dale [5] and Lindqvist et al. [6] in Figure 3. The Larsen score increased during observation in 2 of 3 patients who demonstrated high titers of anti-CCP. Besides, the Larsen score was already high at the start of observation in the remaining one patient (no. 2). In contrast, the Larsen score did not change in patients whose anti-CCP titer was low or negative. Furthermore, one patient (no. 6), whose anti-CCP titer was high at the start of observation and was not detected in her sera during the succeeding four years, also did not show increase in the Larsen score. These observations confirm that a high anti-CCP titer predisposes patients toward joint destruction progression, and probably suggest that decrease in titers of anti-CCP autoantibodies predicts the suppression of joint damage. Therefore, the clinical course of the patients with high titer of anti-CCP antibody may change by the decrease in their titers. However, these insights raise another problem. Do anti-tumor necrosis factor (TNF)-alpha drugs facilitate control of radiographic outcomes over the long term in patients with RA? Recent studies have reported that treatment with infliximab decreased the serum level of rheumatoid factor (RF) but anti-CCP antibodies did not [7, 8]. Thus, many investigators have highlighted the absence of significant variations in anti-CCP titers in patients treated with anti-TNF-alpha drugs in contrast with the level of RF. To resolve this problem, further prospective studies will be required using patients treated with anti-TNF-alpha drugs.

Finally, the decrease in titers of anti-CCP autoantibodies, even if high titer at baseline, may predict the suppression of joint damage. Serially determined anti-CCP antibodies as well as baseline determination should be considered from the predictable viewpoint.

## Figures and Tables

**Table 1:** The 6-patient profiles in the follow-up study during 5 years.

No.	Age/Sex	Disease duration*	Anti-CCP titer	Treatment

1	50/f	2.2	High	SASP**, MTX***, PSL##
2	65/f	12	High	Bucillamine
3	31/f	5	High	SASP, MTX
4	48/f	6	Negative	Bucillamine
5	51/f	5	Low	Bucillamine
6	46/f	2.5	High-negative	SASP

*disease duration (year) at the start of observation; **salazosulphpiridine; ***methotrexate, ^##^prednisolone.
